# Beyond the hype of big data and artificial intelligence: building foundations for knowledge and wisdom

**DOI:** 10.1186/s12916-019-1382-x

**Published:** 2019-07-17

**Authors:** Josip Car, Aziz Sheikh, Paul Wicks, Marc S. Williams

**Affiliations:** 1 Centre for Population Health Sciences (CePHaS), Lee Kong Chian School of Medicine, Nanyang Technological University Singapore, Clinical Sciences Building, 11 Mandalay Road, Singapore, 308232 Singapore; 2 The Usher Institute, The University of Edinburgh, Edinburgh, EH8 9DX Scotland, UK; 3 PatientsLikeMe, 160 Second Street, Cambridge, MA 02142 USA; 4 Genomic Medicine Institute, Geisinger, 100 North Academy Avenue, Danville, PA 17822 USA

**Keywords:** Big data, Electronic health records, Artificial intelligence, Internet of things, Digital health, Genomics, Data sharing, Data privacy, Ethics

## Abstract

Big data, coupled with the use of advanced analytical approaches, such as artificial intelligence (AI), have the potential to improve medical outcomes and population health. Data that are routinely generated from, for example, electronic medical records and smart devices have become progressively easier and cheaper to collect, process, and analyze. In recent decades, this has prompted a substantial increase in biomedical research efforts outside traditional clinical trial settings. Despite the apparent enthusiasm of researchers, funders, and the media, evidence is scarce for successful implementation of products, algorithms, and services arising that make a real difference to clinical care. This article collection provides concrete examples of how “big data” can be used to advance healthcare and discusses some of the limitations and challenges encountered with this type of research. It primarily focuses on real-world data, such as electronic medical records and genomic medicine, considers new developments in AI and digital health, and discusses ethical considerations and issues related to data sharing. Overall, we remain positive that big data studies and associated new technologies will continue to guide novel, exciting research that will ultimately improve healthcare and medicine—but we are also realistic that concerns remain about privacy, equity, security, and benefit to all.

## Introduction

More than ever, medicine now aims to tailor, adjust, and personalize healthcare to individuals’ and populations’ specific characteristics and needs—predictively, preventively, participatorily, and dynamically—while continuously improving and learning from data both “big” and “small.” Today, these data are increasingly captured from data sources both old (such as electronic medical records, EMR) and new (including smartphones, sensors, and smart devices). Combining artificial intelligence (AI) with augmented human intelligence, these new analytical approaches enable “deep learning health systems” that reach far beyond the clinic to forge research, education, and even care into the built environment and peoples’ homes.

The volume of biomedical research is increasing rapidly. Some is being driven by the availability and analysis of big data—the focus of this collection. Despite this, only a tiny fraction of research ever translates into routine clinical care. An analysis by the USA’s Institute of Medicine (now the National Academy of Medicine) noted that it takes 17 years for 14% of research findings to move into clinical practice [1]. As noted by Westfall et al., many factors can affect implementation—several of which involve the use of data. More and more data are generated in medicine, such that big data approaches previously used in fields such as physics and astronomy are increasingly relevant in medicine.

Data, while necessary, are insufficient to inform medical practice. Data must be transformed before it can be useful. A commonly used framework is the “data, information, knowledge, and wisdom” (DIKW) hierarchy. References to this hierarchy date back to the late 1980s in the works of Zeleny [2] and Ackoff [3]. The first reference to this, in the context of medicine, was in the discipline of nursing informatics [4]. This framework was recently revisited by Damman [5], who proposed that the framework be modified to “data, information, evidence, and knowledge” (DIEK) to reflect the importance of evidence. In this framework, “knowledge” is used to denote evidence that is relevant, robust, repeatable, and reproducible. Whichever conceptual framework is preferred, it is evident that data must be transformed to be useful. Despite predictions of the value that big data analytics holds for healthcare [6], medicine has lagged behind other industries in the application of big data to realize its value. Lee and Yoon [7] identify several limitations that affect the use of big data in the medical setting. These include the inherent “messiness” of data collected as a part of clinical care, missing values, high dimensionality, inability to identify bias or confounding, and the observational nature of the data decreasing the ability to infer causality.

The *Beyond Big Data to New Biomedical and Health Data Science* article collection published in *BMC Medicine* focuses on providing examples of how big data-driven approaches might ultimately improve healthcare provision and health outcomes. In addition, the collection’s articles address data complexity, challenges facing this type of research, and other enablers and barriers.

## At the heart of precision health

The dynamism of progress enabled by new data sources is significant. For example, a smartphone microphone within a bedroom environment can now listen for unique gasping sounds, called agonal breathing, which occur when the heart stops beating [8]. These are an audible biomarker—a sign of cardiac arrest and brainstem reflex that arises in the setting of severe hypoxia. An AI algorithm can differentiate them from other types of breathing, with a potential for calling for early cardiopulmonary resuscitation (CPR).

In this article collection, a Debate by Hekler et al. [9] helpfully presents a complementary “small data” paradigm of N-of-1 unit (i.e., a single person, clinic, hospital, healthcare system, community, and city). The authors argue that using these “small data” complements use of big data for advancing personalized medicine, but is also valuable in its own right.

Next, Mackey et al. [10] explore the role of blockchain in use cases such as precision health, drug supply chain, and clinical trials. The authors highlight that beyond the benefits of a distributed, immutable, transparent, and higher trust system, the unique benefits of the much-hyped blockchain for healthcare processes over other existing technologies must be assessed. It is argued that the necessity to share data throughout the ecosystem is what makes blockchain a viable application for healthcare. Healthcare blockchain is, however, not yet “fit-for-purpose,” because it lacks technical data standards and regulatory policies, among other things. The authors have proposed a design framework and set of principles relating to blockchain to help advance the field.

Huang et al. [11] provide a timely reminder that cutting-edge advances in precision health, mHealth, and the use of apps to empower people with diabetes to self-manage their health and disease cannot be achieved without building on sound foundations of evidence-based medicine, following best practices and guidelines. New advances in digital health need quality standards, quality and safety assurance mechanisms, and—at times—even regulation to (counterintuitively for some) speed their adoption.

## Implementation science and genomic medicine

Implementation science is the scientific study of methods to promote the systematic uptake of research findings and other evidence-based practices into routine practice and, hence, to improve the quality and effectiveness of health services and care [12]. Implementation of new findings in genetics and genomics is subject to the same limitations as noted in the introduction, although it is magnified because genomic information is used to define smaller and smaller subgroups of patients—ultimately down to the level of the individual.

Development of the methods of implementation science and the incorporation of implementation science frameworks such as RE/AIM (Reach, Effectiveness, Adoption, Implementation, and Maintenance) [13], the Consolidated Framework for Implementation Research (CFIR), and others [14] has led to great progress in understanding what is needed to implement important research findings into clinical settings. Increasingly, funding agencies are explicitly including the requirement to study implementation, as evidenced by the USA’s National Institutes of Health’s identification of Dissemination and Implementation Science as a research priority [15].

Despite the importance of implementing new findings, the distribution of research funding allocated to data generation compared to that allocated to translation disproportionately favors discovery. For instance, Khoury et al., in an analysis of the genomic translation research continuum from 2007, noted that less than 3% of research publications presented results of T2 research (assessing the value of a genomic application for health practice leading to the development of evidence-based guidelines), with a much smaller proportion devoted to T3 (research to move evidence-based guidelines into health practice, through delivery, dissemination, and diffusion research) or T4 (research that seeks to evaluate the “real-world” health outcomes of a genomic application in practice) research [16]. This has been seen in other areas of biomedical research, and though some improvement has been seen, most publications describe discovery research. To address this issue, one major funder of genetic and genomic research, the National Human Genome Research Institute, explicitly includes implementation research as part of their strategic plan [17].

In this collection, the paper by Namjou et al. [18] emphasizes discovery and implementation—the Electronic Medical Records in Genomics (eMERGE) Network. Namjou and colleagues describe a genome-wide association study (GWAS) looking at non-alcoholic fatty liver disease (NAFLD). What makes this paper exemplary for implementation is the use of natural language processing (NLP) of actual EMR clinical notes to develop a much richer phenotype for discovery than the typical GWAS, which depends heavily on diagnosis codes, a known limitation of these types of studies [19]. eMERGE has been a leader in the development of standardized phenotypes that can be used across EMR systems with high sensitivity and specificity [20]. These phenotypes are available for general use at PheKB.org [21]. The study replicated the known association of NAFLD severity with the *PNPLA3* gene cluster and identified two novel associations: one associated with NAFLD (near *IL17RA*) and another associated with NAFLD progression to fibrosis (near *ZFP90-CDH1*). This study also includes a phenome-wide association study (PheWAS). In contrast to a GWAS, in which a phenotype is tested in cases and controls to identify the genetic loci associated with the phenotype, a PheWAS study tests a known genetic locus in carriers and non-carriers across all phenotypes contained in a health record to discover disease associations with the genetic marker [22]. The PheWAS identified a novel negative association for gout, using the *PNPLA3* gene cluster locus. This study exemplifies how analysis of the big data associated with EMR systems can facilitate discovery, with relevance to real-world disease, and provides an avenue for discovery, dissemination, and implementation.

## Increasing the validity of risk progression models derived from electronic health record-derived data

The drive towards so-called P4 medicine—that is, medicine that is “predictive, preventive, personalized and participatory” [23]—supported by the accompanying increasing availability of EMR-derived clinical cohorts, has led to a proliferation in the development of risk prediction models. Given the very high global disease burden of ischemic heart disease and stroke [24, 25], it is unsurprising that development of cardiovascular risk prediction models has been a major research focus of interest. In a related vein, there has been a policy drive to embed such models into routine clinical care.

In the UK, the National Institute for Health and Care Excellence (NICE) currently recommends use of the QRISK 2 cardiovascular disease algorithm [26]. Using the internationally respected Clinical Practice Research Datalink (CPRD), linking primary care, secondary care, and mortality data, Pate and colleagues [27] constructed a cohort of 3.79 million patients and then tracked risk scores over a 10-year period. They compared the QRISK 2 and 3 algorithms with the incorporation of additional data on secular trends, geographical variation, and approach to imputing missing data. They found that incorporating these additional variables resulted in substantial variation in risk across models. The authors concluded that modeling decisions could have a major impact on risk estimates, particularly secular trends that can relatively easily be accounted for in the modeling process**.**

## Big data, shared data, good data?

While modern technology allows the collection and analysis of data at ever greater scales, the potential for benefit from widespread sharing of data remains hampered by human conventions such as interdisciplinary politics, funding mechanisms, institutional policies, and perverse incentives for career researchers [28], among other research challenges [29]. From the public perspective, there are also potential concerns around fairness, ethics, information governance, and the entry of commercial industries into some health systems. While patients might reasonably assume that medical research professionals routinely and freely share data with fellow academic researchers (and perhaps even industry) on a global scale, they would likely be surprised to hear that most of us do not [30].

Sharing clinical trial data is becoming increasingly commonplace—championed by initiatives such as AllTrials, and demanded by calls from the National Academy of Medicine, the World Health Organization, and the Nordic trial alliance [31]—though it is the oft-criticized commercial sponsors that share more data than their academic counterparts [32]. The landscape of data sharing in practice remains fractured, with a recent review of top biomedical journal practice revealing a split between journals with no formal policy, those that require sharing on request, and those that require full data availability with no restriction [33].

In this collection, Waithira and colleagues [34] argue for clear institution-level policies around data sharing, particularly in low- and middle-income countries. Formal procedures around issues like cost-recovery are particularly important given the lower resource availability in such settings, but also the potential for inequity, given the authors’ experience that most requests to access data from low- and middle-income countries comes from higher-income countries. While the case for data sharing in support of replication, secondary post hoc analysis, and meta-analyses is clear, sharing must not further disadvantage those in the poorest institutions to further the careers of their peers in richer countries.

Ethical considerations around big data sets are also the focus of this collection’s Opinion from Nebeker and Torous [35], who outline ways in which the rapidly evolving landscape of technology presents new and volatile challenges. Ethical frameworks and procedures developed half a century ago for controlled experiments in universities and hospitals struggle when faced with real-time analysis, productization, and monetization of the incalculable “data exhaust” we produce each day with our digital devices. They highlight a newer framework that seeks to balance risks and benefits (as is standard), but also elevates the growing considerations of privacy, data management, access, and usability. The piece serves as a call to action to develop a new digitally minded ethical infrastructure to address these new challenges before the pace of developments in AI, the scale of the “big tech” companies, and the influx of new stakeholders from countries without a robust history of medical ethics, overwhelm our ability to maintain the key principles of justice, beneficence, and respect for persons.

## Conclusions

The United Nations recently reported that, for the first time, half of humanity is now connected to the Internet [36], with major growth in Africa and economically developing countries. Such vast growth in data and connectivity holds great opportunities to gather data, test interventions, and hone care pathways in timescales once thought impossible. Yet, in moving towards an always-online and all-digital culture, we risk forgoing the hard-fought lessons of traditional research. All too often, human bias, generalizability, conflicts of interest, politics, and prejudice still lurk behind the 1s and 0s and the deus ex machina of artificial intelligences that could render simple our complex challenges. While there remains much work to be done, we are cautiously optimistic that we might soon be past the “peak of inflated expectations”, and the “trough of disillusionment” in the so-called “hype cycle” for big data [37]. As this pervasive mega-trend touches off a variety of new technologies and approaches, the foundational work on validity, data sharing, generalizability, and ethical principles described in this special issue will continue to resonate for decades to come.

## Data Availability

Not applicable.

## Abbreviations

AI: Artificial intelligence
eMERGE: Electronic medical records in genomics
EMR: Electronic medical records
GWAS: Genome-wide association studies
NAFLD: Non-alcoholic fatty liver disease
PheWAS: Phenome-wide association study

## References

[1] Westfall JM, Mold J, Fagnan L (2007). Practice-based research: ‘blue highways’ on the NIH roadmap. JAMA..

[2] Zeleny M (1987). Management support systems: towards integrated knowledge management. Hum Syst Manage.

[3] Ackoff RL (1989). From data to wisdom. J Appl Syst Anal.

[4] Matney S, Brewster PJ, Sward KA, Cloyes KG, Staggers N (2011). Philosophical approaches to the nursing informatics data-information-knowledge-wisdom framework. ANS Adv Nurs Sci.

[5] Dammann O (2019). Data, information, evidence, and knowledge: a proposal for health informatics and data science. Online J Public Health Inform.

[6] Murdoch TB, Detsky AS (2013). The inevitable application of big data to health care. JAMA..

[7] Lee CH, Yoon HJ (2017). Medical big data: promise and challenges. Kidney Res Clin Pract.

[8] Chan J, Rea T, Gollakota S, Sunshine JE (2019). Contactless cardiac arrest detection using smart devices. NPJ Digit Med.

[9] Hekler EB, Klasnja P, Chevance G, Golaszewski NM, Lewis D, Sim I. Why we need a small data paradigm. BMC Med. 2019. 10.1186/s12916-019-1366-x. 10.1186/s12916-019-1366-x PMC6636023 31311528

[10] Mackey TK, Kuo T-T, Gummadi B, Clauson KA, Church G, Grishin D (2019). ‘Fit-for-purpose?’ – challenges and opportunities for applications of blockchain technology in the future of healthcare. BMC Med.

[11] Huang Z, Lum E, Jimenez G, Semwal M, Sloot P, Car J. Medication management support in diabetes: a systematic assessment of diabetes self-management apps. BMC Med. 2019. 10.1186/s12916-019-1362-1. 10.1186/s12916-019-1362-1 PMC6636047 31311573

[12] Eccles MP, Mittman BS (2006). Welcome to Implementation Science. Implement Sci.

[13] Glasgow RE, Harden SM, Gaglio B, Rabin B, Smith ML, Porter GC (2019). RE-AIM planning and evaluation framework: adapting to new science and practice with a 20-year review. Front Public Health.

[14] Birken SA, Powell BJ, Presseau J, Kirk MA, Lorencatto F, Gould NJ (2017). Combined use of the Consolidated Framework for Implementation Research (CFIR) and the Theoretical Domains Framework (TDF): a systematic review. Implement Sci.

[15] National Institutes of Health Office of Disease Prevention. Dissemination & implementation (D&I) research. https://prevention.nih.gov/research-priorities/dissemination-implementation. Accessed 1 July 2019.

[16] Khoury MJ, Gwinn M, Yoon PW, Dowling N, Moore CA, Bradley L (2007). The continuum of translation research in genomic medicine: how can we accelerate the appropriate integration of human genome discoveries into health care and disease prevention?. Genet Med.

[17] Green ED, Guyer MS, National Human Genome Research Institute (2011). Charting a course for genomic medicine from base pairs to bedside. Nature..

[18] Namjou B, Lingren T, Huang Y, Parameswaran S, Cobb BL, Stanaway IB, et al. GWAS and enrichment analyses of non-alcoholic fatty liver disease identify new trait-associated genes and pathways across eMERGE network. BMC Med. 2019. 10.1186/s12916-019-1364-z. 10.1186/s12916-019-1364-z PMC6636057 31311600

[19] Robinson JR, Wei W, Roden DM, Denny JC (2018). Defining phenotypes from clinical data to drive genomic research. Annu Rev Biomed Data Sci.

[20] Newton KM, Peissig PL, Kho AN, Bielinski SJ, Berg RL, Choudhary V (2013). Validation of electronic medical record-based phenotyping algorithms: results and lessons learned from the eMERGE network. J Am Med Inform Assoc.

[21] PheKB. https://www.phekb.org/. Accessed 29 June 2019.

[22] Denny JC, Bastarache L, Roden DM (2016). Phenome-wide association studies as a tool to advance precision medicine. Annu Rev Genomics Hum Genet.

[23] Hood L, Friend SH (2011). Predictive, personalized, preventive, participatory (P4) cancer medicine. Nat Rev Clin Oncol.

[24] GBD 2017 DALYs and HALE Collaborators (2018). Global, regional, and national disability-adjusted life-years (DALYs) for 359 diseases and injuries and healthy life expectancy (HALE) for 195 countries and territories, 1990–2017: a systematic analysis for the Global Burden of Disease Study 2017. Lancet.

[25] GBD 2017 Causes of Death Collaborators (2018). Global, regional, and national age-sex-specific mortality for 282 causes of death in 195 countries and territories, 1980–2017: a systematic analysis for the Global Burden of Disease Study 2017. Lancet.

[26] Hippisley-Cox J, Coupland C, Vinogradova Y, Robson J, Minhas R, Sheikh A (2008). Predicting cardiovascular risk in England and Wales: prospective derivation and validation of QRISK2. BMJ..

[27] Pate A, Ernsley R, Ashcroft DM, Brown B, van Staa T. The uncertainty with using risk prediction models for individual decision-making: an exemplar cohort study examining the prediction of cardiovascular disease in English primary care. BMC Med. 2019. 10.1186/s12916-019-1368-8. 10.1186/s12916-019-1404-8 PMC6689154 31399095

[28] Smith R, Roberts I (2016). Time for sharing data to become routine: the seven excuses for not doing so are all invalid. F1000Res..

[29] Kostkova P, Brewer H, de Lusignan S, Fottrell E, Goldacre B, Hart G (2016). Who owns the data? Open data for healthcare. Front Public Health.

[30] Tenopir C, Allard S, Douglass K, Aydinoglu AU, Wu L, Read E (2011). Data sharing by scientists: practices and perceptions. PLoS One.

[31] Loder E., Groves T. (2015). The BMJ requires data sharing on request for all trials. BMJ.

[32] Goldacre B, DeVito NJ, Heneghan C, Irving F, Bacon S, Fleminger J (2018). Compliance with requirement to report results on the EU clinical trials register: cohort study and web resource. BMJ..

[33] Barbui C (2016). Sharing all types of clinical data and harmonizing journal standards. BMC Med.

[34] Waithira N, Mutinda B, Cheah PY (2019). Data management and sharing policy: the first step towards promoting data sharing. BMC Med.

[35] Nebeker C, Torous J, Bartlett Ellis RJ. Building the case for actionable ethics in digital health research supported by artificial intelligence. BMC Med. 2019. 10.1186/s12916-019-1377-7. 10.1186/s12916-019-1377-7 PMC6636063 31311535

[36] More than half of global population now online: UN. Globe post; 2017. https://theglobepost.com/2018/12/07/half-of-population-online/. Accessed 27 June 2019.

[37] Woodie A. Why Gartner dropped big data off the hype curve. Datanami; 2015. https://www.datanami.com/2015/08/26/why-gartner-dropped-big-data-off-the-hype-curve/. Accessed 27 June 2019.

